# Multidisciplinary Clinical Management of a Localized Aggressive Periodontitis diagnosed in a Child with Glanzmann’s Thrombasthenia

**DOI:** 10.5005/jp-journals-10005-1536

**Published:** 2018-08-01

**Authors:** Tony Prud’homme, Elisabeth Roy, Assem Soueidan, Marc Fouassier, Sylvie Dajean-Trutaud, Zahi Badran

**Affiliations:** 1 Surgeon, Department of Pediatric Dentistry, University of Nantes Nantes, France; 2 Surgeon, Department of Pediatric Dentistry, University of Nantes Nantes, France; 3 Professor, Department of Periodontology, University of Nantes, Nantes France; 4 Surgeon, Department of Hematology/Biology, University Hospital of Nantes, Nantes, France; 5 Surgeon, Department of Pediatric Dentistry, University of Nantes Nantes, France; 6 Professor, Department of Periodontology, University of Nantes, Nantes France; Faculty of Dentistry, McGill University, Montreal, Canada

**Keywords:** Aggressive periodontitis, Glanzmann’s thrombasthenia, Periodontal disease/therapy, Periodontopathogen.

## Abstract

Localized aggressive periodontitis (LAP) in child involving primary dentition is a rare disease. The main characteristics of LAP are deep periodontal pockets, bone loss, tooth mobility, and, sometimes, spontaneous tooth loss. The LAP involves only some specific teeth. Glanzmann’s thrombasthenia (GT) is a rare autosomal recessive bleeding disorder. The paper’s aim is to present the case of a 5-year-old girl with GT presenting LAP, and discuss her clinical management.

**How to cite this article:** Prud’homme T, Roy E, Soueidan A, Fouassier M, Dajean-Trutaud S, Badran Z. Multidisciplinary Clinical Management of a Localized Aggressive Periodontitis diagnosed in a Child with Glanzmann’s Thrombasthenia. Int J Clin Pediatr Dent 2018;11(4):344-348.

## INTRODUCTION

Periodontal diseases (PD) are characterized by an imbalance between periodontal pathogens and host defenses, thus causing an inflammatory reaction around teeth supporting tissues.^1^ Aggressive periodontitis is the less common form of PD. Also, development of aggressive periodontitis in primary dentition is rather rare.

Before the “International Workshop for a Classification of Periodontal Diseases and Conditions” held in 1999, such periodontal conditions were classified in the “early-onset periodontitis” family and more specifically as pre-pubertal periodontitis.^2,3^ Periodontal diseases in children were then called aggressive periodontitis, and can be found in primary, mixed, and permanent dentition. Furthermore, they can be separated into two groups according to their localization: Localized or generalized form.^1^ The frequency of aggressive periodontitis in primary dentition is difficult to estimate. Prevalence ranging from 0.9 to 4.5% has been found in different studies, but these figures included all forms of periodontitis in the primary dentition.^4-6^

Localized aggressive periodontitis was found to be strongly associated with the presence of *Aggregatibacter actinomycetemcomitans* (Aa).^7,8^ Many studies have also suggested that family history of LAP points to genetic transmission of such clinical forms. Thus, it seems to be also associated with monocyte deficiency, such as disrupted neutrophil differentiation or chemotaxis.^9^ Furthermore, the affected patients have been shown to have inflammatory hyperresponsive phenotype.^10^ This genetic predisposition to disease is also found in the expression of major histocompatibility complex, human leukocyte antigen (HLA) genes or cytokines. However, available data indicate that Aa presence and early bone loss in primary dentition do not necessarily predispose individuals to periodontal attachment loss in permanent dentition,^11^ and likewise, children treated for LAP do not always exhibit recurrence of PD even in the absence of supportive periodontal therapy (SPT).^12^

Nevertheless, LAP has to be managed by early detection, suppression of the pathogenic flora by mechanical/ antiseptic therapy and potential use of systemic antibiotics. After the initial cause-related therapy, proper SPT is then essential.^1^ The LAP in primary dentition has to be treated as soon as diagnosed, to avoid the risks of becoming a generalized aggressive periodontitis.^1^

Glanzmann’s thrombasthenia is presented as an autosomal recessive and severe bleeding disorder. The latter is seen in children and characterized by impaired primary hemostasis;^13^ αIIb and β3 genes could be both concerned. Integrin synthesis depends on αIIbβ3 complex formation and is then directly affected, because incorrectly folded or noncomplexed gene products are rapidly degraded. Furthermore, GT is characterized by defect of the platelet membrane glycoprotein IIb to IIIa complex, causing defective platelet hemostatic plug formation, thus leading to severe bleeding. The recurrent oral manifestation of GT is gingival bleeding (GB). The disease is reported to be especially prevalent in populations where intermarriage is common.^13^ Although GB is often described in cases of GT, there are, to the best of our knowledge, no described cases of GT-associated LAP.

**Figs 1A and B:**
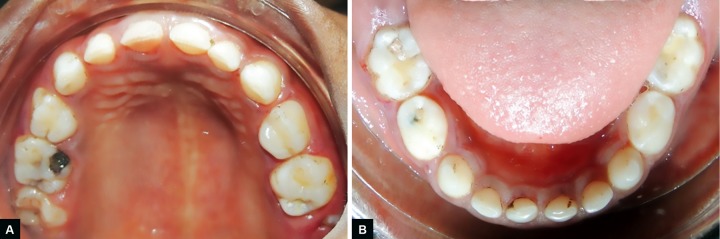
Initial clinical examination of the patient. (A) Maxillary and (B) mandibular aspects

This report presents and discusses the clinical management of a young girl diagnosed positively for both LAP and GT.

## CASE REPORT

A 5-year-old African girl presented to Dental Care Unit of the University hospital of Nantes for a general dental check-up, her sister having been previously diagnosed with LAP. Clinical examination showed no visible signs of gingival inflammation and no pain-related complaints were expressed by the patients.

**Figs 2A to D:**
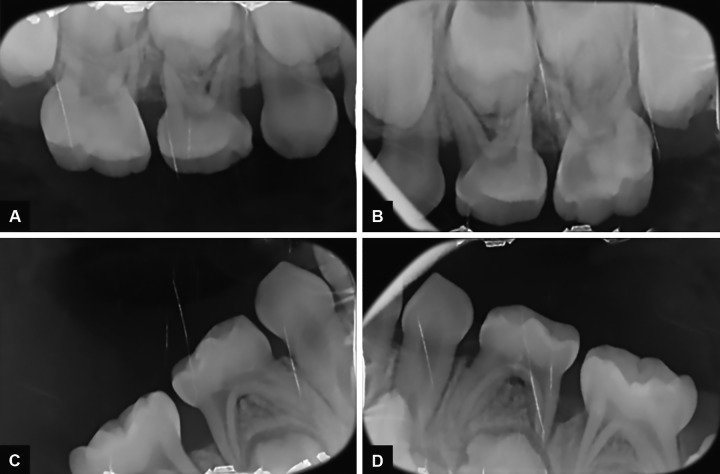
Initial radiographic examination of the patient

Medical history of the patient revealed a GT associated with a slight anemia. She presented a complete primary dentition and the first permanent maxillary molars were partially erupted (Fig. 1). Oral hygiene was poor and plaque deposits were present around all teeth. Black stains were also noticed. Carious lesions could be found on the occlusal surface of multiple primary molars (#: A, J, L, K, and T). Radiographic examination showed severe horizontal bone loss around the four first primary molars (#: B, I, L, and S) (Fig. 2). Clinical examination of these teeth revealed the presence of severe periodontal attachment loss and slight mobility increase. Periodontal probing depths were assessed and pockets depth of 5 mm around affected teeth.

**Figs 3A and B:**
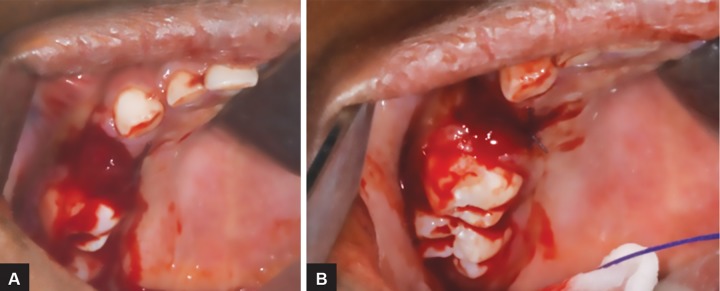
Extraction and hemostasis management of 54

The treatment goal was to avoid further progression of the bone loss to the rest of the dentition and particularly permanent teeth. Therefore, it was decided to extract all the first primary molars affected by severe periodontitis, and to proceed on a thorough scaling and root planing on all the other teeth.

Due to the patient age and cooperation, her treatment was conducted under general anesthesia, and consisted of the extraction of the four affected teeth (Fig. 3). Management of carious lesions was also done at this occasion. She was injected slowly (5 minutes) with Eptacog alpha activated (rh-FVIIa, Novoseven^®^, Novo Nordisk, France) right before the surgery and then 2 hours after the first injection. All necessary means for blood hemostasis were also used during surgery:

- Sutures (Vicryl 4.0)
- Hemostatic sponge (Pangen^®^, Urgo, France)
- Biological glue (Tissucol^®^, Baxter, France)

She also benefited from a tranexamic acid (Exacyl^®^ Sanofi-Aventis, France) prescription, started the evening of the surgery and continued for 10 days, 100 mg three times a day for 10 days.

Extracted teeth showed no signs of root resorption. After surgery, she was treated with systemic antibiotics for 7 days with two daily prescriptions of amoxicillin (50-100 mg/kg/day). No antibiotic-related side effects were reported and the parents supervised medication intake over the whole period.

One week after the surgery, the patient did not complain of any pain or discomfort. Satisfactory wound healing was achieved without complications. Intraoral hygiene was adequate, and parents were reinstructed to watch it.

Both parents were also screened for periodontal disease. The mother exhibited a slight gingivitis and was referred for periodontal treatment. The older sister (OS) and brother were also screened, and although the 3-year-old brother presented no signs of periodontal disease, the 8-year-old sister showed periodontal breakdown with no diagnosed contributory medical history.

The OS had mixed dentition with the first permanent molars, the permanent mandibular incisors, and the central maxillary incisors fully erupted. Oral hygiene was satisfactory. Radiographic (Fig. 4) and clinical examination permitted the establishment of LAP diagnosis. No sign of bone loss around permanent teeth could be detected. There was also a shallow carious lesion on the 84. Proper dental and periodontal management was delivered.

## FOLLOW-UP

The follow-up period was 9 months until now, with a recall visit scheduled every 3 months. Periodontal prophylaxis and oral hygiene motivation were carried at each visit. Clinical and radiographic examination revealed no signs of a periodontal disease recurrence (Fig. 5).

The OS had the same supportive periodontal treatment protocol. The same satisfactory outcome was observed at each visit. The first permanent premolar erupted uneventfully.

## DISCUSSION

Case of severe periodontal destruction in young children is rare and mostly associated with systemic diseases. Only few reports of LAP affecting the primary dentition without history of systemic disease^14,15^ could be found in the literature. When applying the exact definition of LAP, first permanent molars have to be affected.^2^ We chose to speak of LAP on primary dentition, as previous authors did, to simplify the denomination of the observed periodontal disease. The GT does not present any disturbance of host defense and does not justify to qualify the described case as a “Periodontitis due to Systemic pathology,” especially that OS presents the same periodontal condition with no evident contributory medical history.

Periodontal diseases management consists of prevention, followed by early detection and treatment, thus achieving adequate periodontal pathogens elimination and infection control. In case of LAP in primary dentition, there is a strong possibility of infection spread to permanent dentition.^1^ When LAP diagnosis is confirmed, clinical therapy should be established quickly.

**Fig. 4A:**
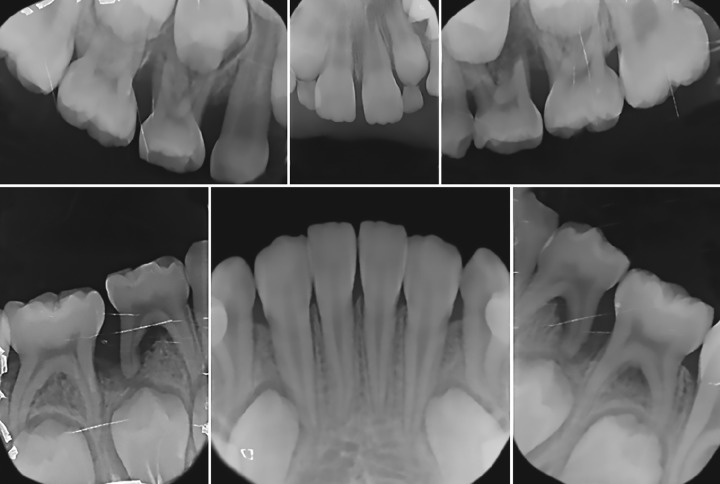
Radiographic situation before therapy of the OS (Periapical radiography)

**Fig. 4B:**
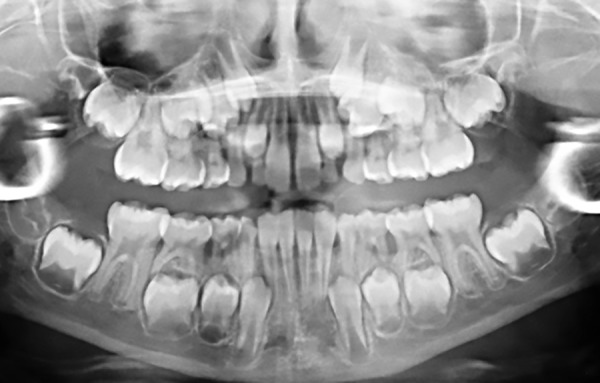
Radiographic situation before therapy of the OS (Orthopantomogram)

**Figs 5A to D:**
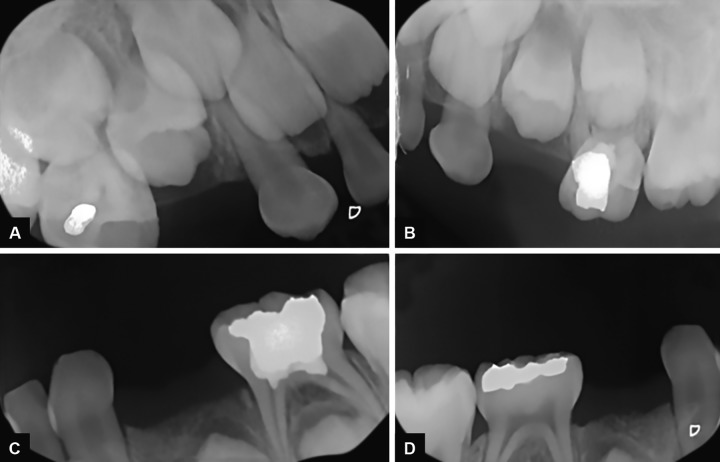
Radiographic situation 3 months after therapy

For successful treatment outcome, clinical decision-making should be adapted to the patient’s status/needs. Success key factor would be patient compliance. Disease progression and location, pediatric and orthodontic necessity, and, eventually, bacterial composition should also be assessed. For our described patient, we needed general anesthesia, because of her lack of compliance and especially for her bleeding disorder challenge.

We chose a conservative approach and maintained all noninfected primary teeth to preserve functionality and esthetics, in accordance with published clinical protocols.^14,15^ Suggestion was made for space-maintainer but the parents refused due to financial issue.

The LAP was observed in association with GT for the first time, to the best of our knowledge. It is not clear if this is a pure coincidence, or if the two pathologies share common genetic predisposition or pathophysiological pathway. We hypothesize that circulatory disorders in GT could interfere with periodontal inflammation process in LAP. On the contrary, GT increases clinical management challenge of LAP cases. Hence, treatment options should take into account GB exacerbation.

Finally, it is obvious that further clinical and biological investigations are necessary to clarify if the described case of GT-associated LAP is an isolated case or if GT-positive patients should be screened systematically for LAP.

## CONCLUSION

This case report described the case of a 5-year-old girl diagnosed for both GT and LAP. The latter was successfully treated with conservative approach.
